# Development of assays for the characterization of sperm motility parameters, viability, and membrane integrity in the epididymis and vas deferens of the greater rhea (*Rhea americana*)

**DOI:** 10.1590/1984-3143-AR2023-0113

**Published:** 2024-01-05

**Authors:** Luana Grasiele Pereira Bezerra, Andréia Maria Silva, Maiko Roberto Tavares Dantas, Romário Parente dos Santos, Samara Sandy Jeronimo Moreira, Ana Glória Pereira, Moacir Franco de Oliveira, Pierre Comizzoli, Alexandre Rodrigues Silva

**Affiliations:** 1 Laboratory of Animal Germplasm Conservation, Department of Animal Sciences, Universidade Federal Rural do Semi-Árido, Mossoró, RN, Brasil; 2 Smithsonian National Zoo and Conservation Biology Institute, Washington, USA

**Keywords:** ratites, sperm pathways, avian sperm, bird semen, wildlife

## Abstract

The objectives of the study were to (1) describe the kinematic parameters of spermatozoa (2) compare methods of evaluating sperm viability (3) validate assays of functionality and integrity of the sperm membrane and (4) evaluate possible changes between spermatozoa from the epididymis and the vas deferens of the greater rhea. Semen samples were recovered from 7 adult individuals. Sperm motility was characterized by adjusting the set-up for Computer-assisted semen analysis (CASA) to that new species. For sperm viability evaluation, smears of bromophenol blue and eosin-nigrosine dyes were used. Five solutions of different osmolarities were then tested for the hypoosmotic swelling test (HOST). The combination of fluorescent probes (propidium iodide - IP and Hoechst 33342) was also used to assess plasma membrane integrity. Data were presented as mean ± SEM. Rhea spermatozoa from the vas deferens had an overall motility of 14.6 ± 2.5%. The bromophenol blue staining technique revealed that 64.6 ± 5.2% sperm were viable, while that proportion was 72.1 ± 2.5% using eosin-nigrosine. An average of 77.6 ± 4.8% of spermatozoa reacted to the HOST with distilled water at 0 mOsm/l. Fluorescent probes indicated that 65.3 ± 2.6% of spermatozoa had intact membranes. Interestingly, no statistical differences were observed between the parameters analyzed in the epididymal spermatozoa and the vas deferens. These new assays set reference values that can now be used to further exploration of sperm handling conditions and freezing protocols in rheas.

## Introduction

Ratites are a taxonomic group of flightless birds that play a significant role in seed dispersal and predator regulation, thereby contributing to the ecological balance of their respective ecosystems (Nield et al., 2019). This group comprises the ostrich (*Struthio camelus*), emu (*Dromaius nonaehollandiae*), kiwi (*Apterix sp.*), and rhea (*Rhea americana*) (Sibley and Monroe, 1996). Rheas are indigenous to South America that, according to the classification of the Convention on International Trade in Species of Wild Fauna and Flora in Danger of Extinction (CITES), although they are currently not necessarily in danger of extinction, they could reach this situation (CITES, 2023). Despite their ecological importance, knowledge about these birds remains limited, particularly concerning their reproductive aspects (Phillipis and Asa, 1989; Parizzi et al., 2007; Santos et al., 2011; Góes et al., 2010). Understanding their reproductive biology is crucial for the development of effective conservation strategies.

In a recent study conducted by our research group, we demonstrated that rhea spermatozoa share morphological similarities with gametes of other ratite birds, despite their smaller size and elongated shape. Additionally, we observed a significant increase in the dimensions of these spermatozoa as they traveled from the epididymis to the vas deferens, indicating a maturation process (Bezerra et al., 2023a). However, to gain a comprehensive understanding of sperm physiology in this species, detailed information regarding the functional parameters of rhea sperm is still lacking. In this context, contemporary research suggests that avian sperm gradually acquire the necessary fertilization abilities while passing through the male reproductive tract before ejaculation (Ahammad et al., 2011). These functional modifications in sperm may be associated with changes in motility patterns and alterations in the structure of the plasma membrane. It is well known that during transit in the vas deferens, proteins are secreted and incorporated into the plasma membrane of avian spermatozoa. These proteins serve to protect spermatic cells against oxidative damage and contribute to the essential sperm functions required for fertilization (Ahammad et al., 2011).

A prerequisite for investigating the sperm membrane is to have knowledge of cell motility. Computer-assisted semen analysis (CASA) is a suitable method for obtaining individual sperm motion and kinematic data, thereby enhancing the accuracy of evaluation (Amann and Waberski, 2014). The application of CASA has previously been documented in the examination of sperm from ratites such as emu (Sood et al., 2012) and ostrich (Muvhali et al., 2022). However, in the case of rheas, only subjective assessment of motility has been carried out (Góes et al., 2010; Bezerra et al., 2023a).

The viability of bird sperm cells can be assessed using supravital dyes as the bromophenol blue and the eosin-nigrosine (Madeddu et al., 2009; Sood et al., 2012). These dyes can penetrate spermatozoa that have an injured membrane, allowing to distinguish viable from non-viable sperm cells (Tartaglione and Ritta, 2004).

Different methods can be employed to assess the functionality and structural integrity of the sperm plasma membrane. The Hypoosmotic Swelling Test (HOST) is a technique that relies on the flagellum’s ability to fold when exposed to a hypoosmotic solution. This test indicates whether water transport has occurred across the plasma membrane, thus confirming its functional status (Bezerra et al., 2023b). An ideal hypoosmotic solution should apply sufficient osmotic stress to cause a visible increase in volume, while avoiding lysis of the sperm membrane (Jeyendran et al., 1984). For the HOST test in ostriches, a hypoosmotic solution of 25 mOsm/l has been employed (Smith et al., 2023), while emus have been evaluated using hypoosmotic solutions of 50 mOsm/L (Matson et al., 2009). However, the assessment of sperm membrane functionality in rheas is currently lacking, and it is imperative to identify the optimal solution for conducting test of osmotic tolerance.

The evaluation of structural integrity of the sperm plasma membrane can be performed using vital dyes under optical microscopy, as demonstrated in emus (Sood et al., 2012). Alternatively, contemporary methods highlight the effectiveness of fluorescent dye combinations in identifying membrane damage, as observed in ostriches (Smith et al., 2023). In this regard, the utilization of propidium iodide (PI) and Hoechst 3342 in combination (Bezerra et al., 2023b) appears to be a feasible approach for analyzing the plasma membrane of rhea spermatozoa. Nevertheless, this methodology necessitates validation for the specific species.

The objectives of the study were to (1) describe the kinematic parameters of rhea spermatozoa (2) compare methods of evaluating sperm viability (3) validate assays of functionality and integrity of the sperm membrane and (4) evaluate possible changes between spermatozoa from the epididymis and the vas deferens.

## Material and methods

### Ethical considerations

The ethics committee of the Universidade Federal Rural do Semi-Arido (UFERSA) has approved the experimental protocols as well as the animal care procedures adopted (Process no. 23091.001423/2020-8). In addition, experiments were approved by Brazilian Ministry of the Environment (SISBIO no. 73638-1). The reagents used in this study were obtained from Sigma-Aldrich (St. Louis, MO, USA).

### Animals

The animals were obtained from the Center for the Multiplication of Wild Animals (CEMAS/UFERSA; Scientific register N^°^ 12.492-0004/IBAMA), located in the city Mossoró - Rio Grande do Norte in Brazil, at 5^°^ 11^’^ S and 37^°^ 20^’^ W, at an average altitude of 16 m in a region characterized by a semi-arid tropical climate, presenting Köppen climatic classification as dry and very hot, with an average annual temperature of 27.4°C. The rhea paddocks had a dimension of 20 x 10 m. The birds were fed with concentrate based on corn, wheat bran and soybean, fruits were also provided, and water was provided *ad libitum*. Experiments took place during the breeding seasons (June to December) from 2019 to 2021. We were authorized to cull a small group of individuals at CEMAS/UFERSA in order to promote population control, from which animals could be used for scientific experimentation. Seven adult rheas in reproductive age of 2.9 ± 0.4 (range 1.5 – 5.0) years old and with an average weight of 23.1 ± 1.1(range 20.3 – 27.2) kg were used.

### Sperm collection

Animals were captured manually and euthanized based on the protocol used by Pollock et al. (2005). After mechanical restraint, the animals were weighed and premedicated with an intramuscular combination of xylazine hydrochloride (Xilazin^®^ 2%, Syntec, Pharmaceutical Technology Applied to Veterinary Medicine, São Paulo, Brazil) and ketamine hydrochloride 15mg/kg (Quetamina^®^, Vetnil, Sao Paulo, Brazil). After reaching the sedation stage, the animals received a dose of Thiopental (Thiopentax; Cristalia, São Paulo, SP, Brazil) intravenously as an anesthesia inducing drug. When the anesthetic plane was reached, the animals were euthanized with an intracardiac administration of Potassium Chloride (Potassium Chloride^®^ 19.1%, Equiplex, Goiânia, GO, Brazil), confirming the animal’s death after identifying the cardiopulmonary arrest.

Epididymis and vas deferent were then collected, wrapped in gauze moistened with 0.9% saline at 38 °C and transported to the laboratory for processing. Subsequently, the testis-epididymis-ductus deferens complexes were dissected, washed externally with physiological solution at 38 °C, weighed and measured. To obtain sperm, the flotation technique was used. Briefly, the epididymis and ducts were separated and sliced with a scalpel in a Petri dish containing saline at 38 ° C. After 5 minutes in a static position, the tissues were removed and the suspension was evaluated (Bezerra et al., 2023a).

### Experimental design

Initially, only sperm samples recovered from the vas deferens were used for the establishment of analyzing methods. These were related to adjusting the CASA set up to evaluate rhea sperm motility and kinematic parameters, as well as testing different hypoosmotic solutions for HOST test, and comparing different vital dyes for viability analysis, and fluorescent probes for the evaluation of the rhea sperm membrane integrity through a flash-frozen assay. Then, we used the established methods to characterize alterations in the rhea sperm from the epididymis to the vas deferens.

### Sperm samples overall evaluations

Immediately after collection, the appearance and color of the samples were observed. The recovered volume was measured using pipettes and graduated tubes. An aliquot of the sample containing spermatozoa (5 μl) was diluted in 10% buffered formalin (995 μl) and the sperm concentration was esteemed using a Neubauer counting chamber visualized in a light microscope (400x) (Nikon Eclipse E200, Nikon Instrument, Tokyo, Japan). The number of recovered spermatozoa was measured, multiplying the sperm volume and concentration (Bezerra et al., 2023a).

### Computer-assisted semen analysis (CASA)

For the analysis of the kinetic parameters, the IVOS 7.4G system (Hamilton-Thorne Research, Beverly, MA, USA) was used for computer-assisted semen analysis (CASA). The samples (3 μl) were placed in the chamber of a Leja slide (ref: SC-20-01-04-B, IMV Technologies), with the tip at 45° and the outer droplet was dried. Once the sample drift was stabilized, five random fields were selected and analyzed. The pre-established CASA configurations for emu (*Dromaius novaehollandiae*) spermatozoa reported by Sood et al. (2012) were used as the basis for the rhea spermatozoa configurations. For that, we used the rhea sperm morphometric dimensions previously described by our team (Bezerra et al., 2023a), and executed the necessary adjustments in the set up following manufacturer assistance. Under technical assistance from the team at the equipment manufacturer Hamilton-Thorne LTD., changes were tentatively made until the system had the highest possible number of cells counted by the CASA system, and we could then establish the best settings for the analysis of the rhea sperm. The following motility characteristics were determined: total motile (MOT; in %), progressive motility (PMOT; in %), path velocity (VAP; in µm/s), progressive velocity (VSL; in µm/s), curvilinear velocity (VCL; in µm/s), lateral amplitude (ALH; in µm), Beat Frequency (BCF; in Hz), Straightness (STR; in %) and Linearity (Lin; in %). According to low VAP cutoff (LVV) and medium VAP cutoff (MVV), the overall sperm population was subdivided into four categories: rapid, with VAP > MVV; medium, with (LVV < VAP < MVV); slow, with VAP < LVV; and static, the proportion of cells that were not moving (Verstegen et al., 2002). For a reliable evaluation of the sperm tracks, the Edit Tracks Option of the IVOS 7.4G system was used to exclude the debris derived from the extenders. A further dilution in salt solution (1:1) was conducted only if necessary.

### Assessment of sperm viability by vital dyes

Two different smears were used for the evaluation of sperm viability using vital dyes according to methodology previously described for hawks (Fischer et al., 2020). The first was made from 10 µl of sample mixed with the same volume of bromophenol blue (1 g of bromophenol blue + 4 g of tribasic sodium citrate + 1 ml of distilled water), and the other using the same proportion of sperm sample and eosin-nigrosin dyes (1 g of yellowish eosin + 2g of nigrosin + 2.941 g of sodium tricitrate 2H_2_O + 100 ml of distilled water). Then, the mixtures were deposited on slides and dragged with the aid of another slide. After drying the slides at room temperature, 200 cells were counted in optical microscopy (400x) (Nikon Eclipse E200, Nikon Instrument, Tokyo, Japan). For spermatozoa stained with bromophenol blue, transparent cells were considered viable, and cells stained in blue were considered non-viable (Fischer et al., 2020). As for spermatozoa stained with eosin-nigrosine, those that presented pink coloration were considered non-viable (Fischer et al., 2020).

### Hypoosmotic swelling test

To validate the hypoosmotic solution for the analysis of plasma membrane functionality, a methodology described for quail sperm was used (Bezerra et al., 2023b). Briefly, aliquots (5 μl) of the samples containing the sperm were added to 45 µl of different hypoosmotic solutions and incubated at 37°C for 40 min to validate the HOST. The hypoosmotic solutions consisted of distilled water (0 mOsm/l) and fructose solutions (50, 100, 150, or 200 mOsm/l). For the preparation of the solutions, a solution based on fructose (50%) at 200 mOsm/l was made and serial dilutions were performed in distilled water, forming media of four different osmolarities: 50, 100, 150 and 200 mOsm/l. After incubation, the sperm were observed under phase-contrast microscopy (x1000). A total of 200 sperm were counted in at least five fields and classified as reactive or non-reactive, based on the presence or absence of curled (swollen) tails, respectively.

### Assessment of plasma membrane integrity using a combination of fluorescent probes

For the validation of the use of fluorescent probes for the evaluation of plasma membrane integrity, a methodology of a flash-frozen assay described for quails by Bezerra et al. (2023b) was used. Briefly, the samples were divided into two aliquots, where one remained viable and the other was subjected to rapid freezing in liquid nitrogen. This was followed by slow defrosting three times to cause damage to the cell membrane. Three treatments were prepared from these aliquots with the following proportions of fresh sperm and sperm subjected to flash freezing: 100:0 (T100), 50:50 (T50), and 0:100 (T0). An aliquot (10 μl) of each treatment was incubated at 37°C for 10 min with 2 μl Hoechst 33342 (H342, Molecular Probes, Eugene, OR, U.S.A) diluted at 25mg/ml in DMSO. Subsequently, 3μl of PI (PI, Thermo Fisher Scientific, Waltham, MA, U.S.A) composed of 25 mg/ml stock solution (25 mg of PI +1 ml of dimethyl sulphoxide; DMSO, Sigma- Aldrich, Burlington, MA, U.S.A) diluted at 2 mg/ml with 80 μl of PI stock solution + 920 μl of phosphate-buffered saline (PBS, Sigma-Aldrich, Burlington, MA, U.S.A), which was added to each sample. The samples were then incubated at 37°C for 8 min. The samples were observed with an epifluorescence microscope (Olympus B×51TF, Tokyo, Japan), with a 100 W mercury discharge burner (U-LH100HG) as the fluorescence light source and analyzed under a yellow filter U-FYW (540–585 nm) by counting 100 cells per sample. Spermatozoa marked in red (PI) were classified as injured membrane. Spermatozoa that emitted only the blue counterstaining (H342) were classified as having an intact plasma membrane.

### Statistical analysis

Data were expressed as mean ± standard error and were tested for normality using the Shapiro-Wilk test and for homoscedasticity using the Levene test. Differences related to hypoosmotic solutions were evaluated by ANOVA, followed by Tukey’s Post-Hoc test. Differences between flash frozen treatments were assessed by Kruskal-Wallis test, followed by Dwass-Steel-Critchlow-Fligner comparisons. Simple linear regression was used to identify correlations among different osmolalities (independent variables) and percentage of membrane functionality (dependent variables) and between different flash frozen treatments (independent variables) and percentage of plasma membrane integrity (dependent variables). Correlations between viability assessed by vital dyes and membrane integrity assessed by fluorescent probes were performed using Pearson’s correlation test. Comparisons between samples retrieved from the epididymis or vas deferens were performed by ANOVA followed by Tukey’s Post-Hoc test. Results were considered significant when *P*<0.05. Analyzes were performed using the Jamovi Software version 2.3.19 (Sydney, Australia).

## Results

### Overall semen evaluations

Samples recovered from the vas deferens were thin and watery in appearance, while those retrieved from the epididymis were aqueous in majority but sometimes containing blood. An average of 314.0 ± 95.9 x 10^6^ spermatozoa were recovered from the vas deferens, while 156.0 ± 61.1 x 10^6^ sperm were retrieved from the epididymis. No significant difference was observed between the number of spermatozoa recovered from the two spermatic pathways *(P* < 0.05).

### Computer-assisted semen analysis (CASA) set up for rhea sperm

CASA was able to identify the vast majority of cells and evaluate them. However, round cells and some artifacts were also present and removed from the evaluations in the system. Table1 shows the CASA set ups adjusted for rhea sperm taking the emus set-up as a basis. Using this set-up, we verified that there were no significant differences related to rhea sperm motion kinematic parameters from the epididymis to the vas deferens as shown in Table 2.

**Table 1:** Adjusted parameter settings of the CASA system for evaluating spermatozoa of rheas (*Rhea americana*) using set-up of emus (*Dromaius novaehollandiae*) as a reference (Sood et al., 2012).

**Kinetic parameters**	**Emus**	**Rheas**
Temperature (°C)	-	37
Frames acquired	45	30
Frame rate (Hz)	60	60
Minimum contrast	25	15
Minimum cell size (pixels)	11	9
Straightness threshold (%)	80	80
Average path velocity (VAP) cutoff (µm/s)	10	10
Cell size (pixels)	4	9
Cell intensity	80	19
Static head size	0.72 to 8.80	0.57 to 8.80
Static head intensity	0.14 to 1.84	0.10 to 3.08
Static elongation	0 to 47	4 to 98
Slow cells motile	NO	NO
Magnification	1.89	1.89
Video frequency	60	60
Bright field	NO	NO
Integrating time (frame)	1	1

**Table 2:** Kinetic parameters of rhea (*Rhea americana*) spermatozoa collected from the epididymis and vas deferens. Values are expressed as Mean ± SEM from 7 individuals.

**Kinetic parameters**	**Epididymis**	**Vas deferens**	**Overall**
MOT (%)	8.0 ± 2.4	14.6 ± 2.5	11.9 ± 1.9
PMOT (%)	1.6 ± 0.7	2.6 ± 0.7	2.2 ± 0.5
VAP (µm/s)	69.2 ± 11.2	63.3 ± 4.0	65.8 ± 5.0
VSL (µm/s)	55.2 ± 10.4	50.7 ± 2.5	52.6 ± 4.3
VCL (µm/s)	108.0 ± 19.6	107.0 ± 7.1	108.0 ± 8.6
ALH (µm)	3.4 ± 1.6	4.5 ± 0.7	4.1 ± 0.8
BCF (Hz)	12.4 ± 5.3	33.8 ± 3.6	24.9 ± 4.3
STR (%)	79.4 ± 3.4	80.8 ± 1.3	80.2 ± 1.5
LIN (%)	54.4 ± 4.7	50.6 ± 1.4	52.2 ± 2.1
Sperm subpopulations			
Rapid	2.6 ± 1.2	4.1 ± 1.2	3.5 ± 0.8
Medium	5.2 ± 2.0	10.4 ± 2.1	8.2 ± 1.6
Slow	26.5 ± 10.1	40 ± 4.1	34.4 ± 5.0
Static	65.5 ± 10.6	45.2 ± 5.7	53.7 ± 6.0

### Cell viability

**Figure 1:**
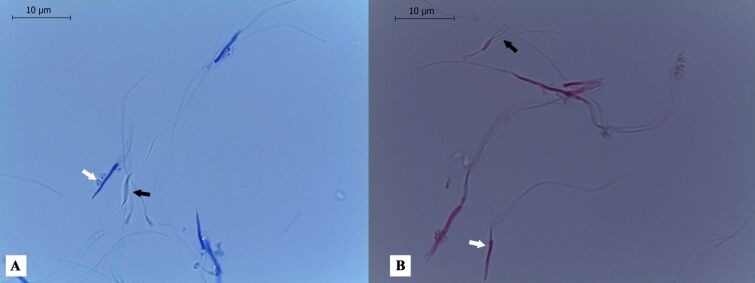
Viability evaluation of Rhea spermatozoa (*Rhea americana*). (A) Spermatozoa evaluated with bromophenol blue dye, black arrow points to viable sperm and white arrow points to non-viable sperm (100x magnification); (B) Spermatozoa evaluated with eosin-nigrosine dye, black arrow points to viable sperm and white arrow points to non-viable sperm (100x magnification).

The evaluation of viability through smears made with bromophenol blue (Figure 1A) and eosin-nigrosine (Figure 1B) proved to be a simple, fast, cheap and field-friendly. However, it was a little subjective, as it presented some color nuances, with some cells strongly stained while others are slightly weaker, which raised doubts at the time of the evaluations. For the staining technique with blue bromophenol, an average of 64.6 ± 5.2% spermatozoa viable were detected, while 72.1 ± 2.5%, were observed for eosin-nigrosine evaluation.

### Plasma membrane functionality

**Figure 2:**
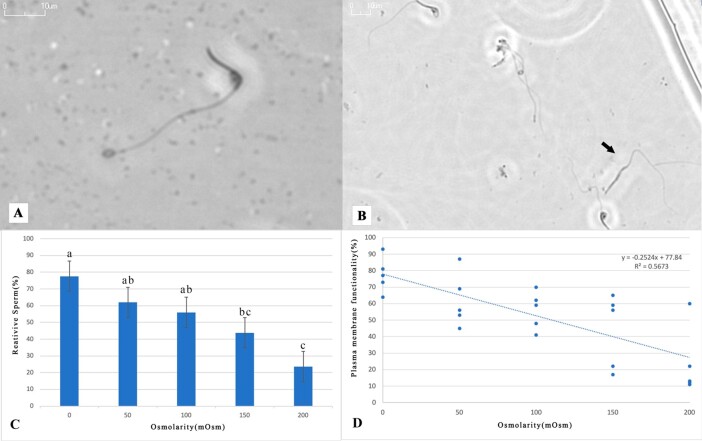
Hypoosmotic swelling test using different hypoosmotic solutions (0, 50, 100, 150 or 200 mOsm/l) for analysis of rhea (*Rhea americana*) sperm membrane functionality. (A) Reacted spermatozoa (80x magnification); (B) Unreacted spermatozoa in the hypoosmotic test (HOST) to assess membrane functionality (black arrow) (40x magnification); (C) Mean values (± SEM) for spermatozoa from reactive sperm after the hyposmootic swelling test; (D) Linear regression showing a moderate correlation between the different osmolarities (0, 50, 100, 150 or 200 mOsm/l) and the percentage of plasma membrane functionality assessed by the hypoosmotic swelling test (dependent variable). ^a,b^Different lowercase letters indicate a significant difference between treatments (P < 0.05).

Reacted spermatozoa (curled tails) and unreacted (straight tails) were observed as shown in Figures 2A and 2B respectively. Regarding the validation of the HOST test, the greatest osmotic responses of rhea sperm (*P* < 0.05) were achieved at 0 mOsm/l (77.6 ± 4.8%), followed by 50 mOsm/l (62.0 ± 7.3%), and 100 mOsm/l (56.0 ± 5.1%), compared to solutions of 150 mOsm/l (43.8 ± 10.1%) or 200 mOsm/l (23.6 ± 9.3%) (Figure 2C). Linear regression analysis showed a moderate correlation (R^2^=0.5673) between the membrane functionality results and the tested hypoosmotic solutions (Figure 2D). Using the 0 mOsm/l solution, we verified that there were no significant differences related to plasma membrane functionality between samples recovered from the epididymis (74.6 ± 4.7%) to the vas deferens (77.6 ± 4.8%).

### Plasma membrane integrity

**Figure 3:**
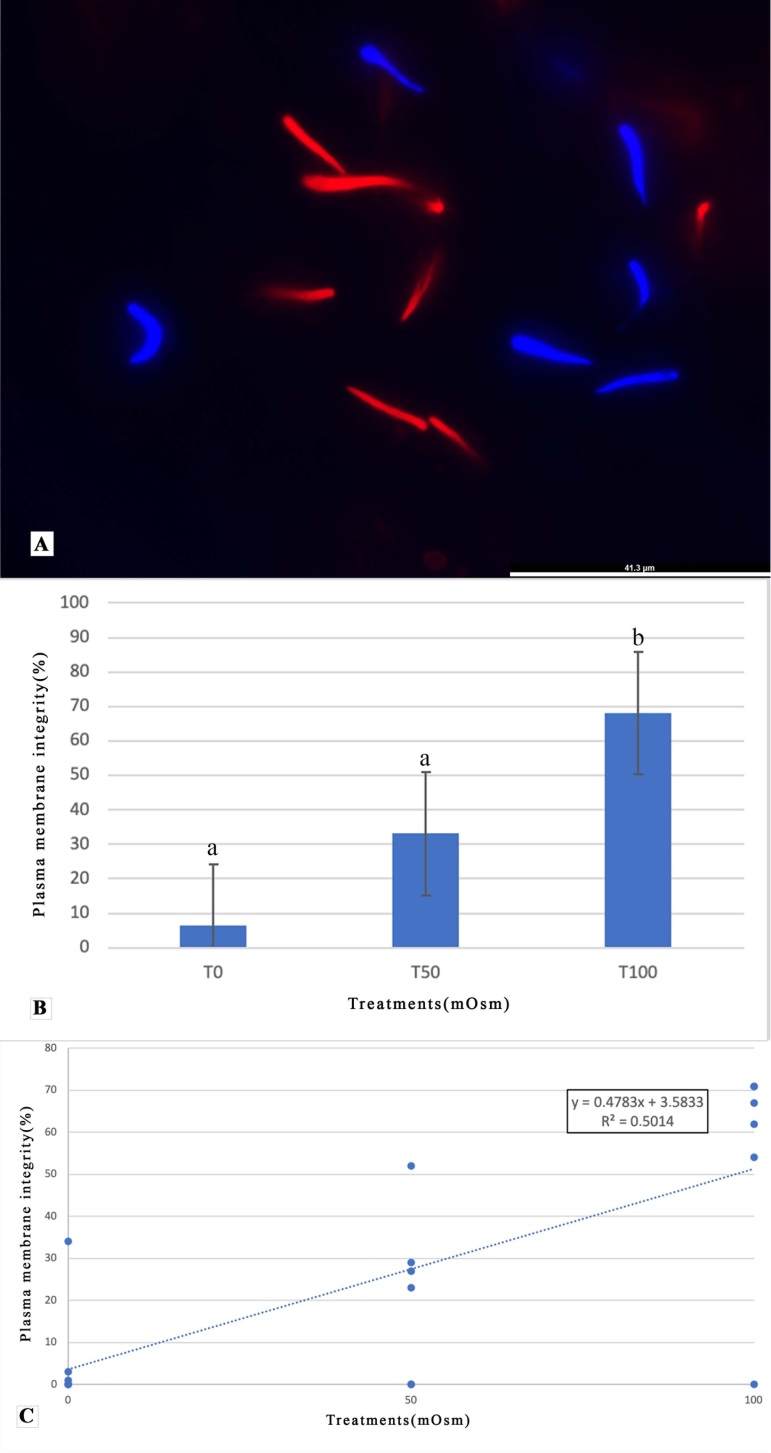
Plasma membrane integrity of rhea (*Rhea americana*) spermatozoa evaluated by the combination of fluorescent probes (Propidium Iodide/Hoechst 3342). (A) Spermatozoa with damaged membrane stained with propidium iodide (PI) and sperm nuclei counterstained with Hoechst 3342 only (100x magnification); (B) Mean values (± SEM) for plasma membrane integrity of rhea sperm. Treatments T0 (100% frozen sperm); T50 (50% fresh sperm plus 50% frozen sperm), and T100 (100% fresh sperm); (C) Linear regression showing moderate correlation between flash frozen treatments (independent variable) and the percentage of plasma membrane integrity assessed by fluorescent probes (dependent variable). ^a,b^Different lowercase letters indicate significant difference between treatments (P<0.05).

The evaluation using fluorescent probes showed intense markings (Figure 3). Spermatozoa with injured membrane showed red markings due to PI penetration (Figure 3A). Spermatozoa with an intact membrane were not marked in red, they only showed the blue counterstaining (H342) (Figure 3A).

The flash frozen results for validating the probes showed that the T100 treatment had the highest value (65.3 ± 2.6%) for sperm with intact plasma membranes (stained in blue). Almost a half of this value (33.1 ± 5.1%) was observed in T50, and 6.3 ± 5.5% was observed in the T0 treatments (Figure 3B). We found significant differences (*P* < 0.05) among the T100, T50, and T0 treatment groups, as shown in Figure 3B. Linear regression showed a moderate correlation (R^2^ = 0.5014) between the percentage of cells with intact membranes and flash frozen test treatments, as displayed in Figure 3C.

Using the fluorescent probes, no significant difference (*P* > 0.05) was observed in the plasma membrane integrity of spermatozoa recovered from the epididymis (74.6 ± 3.5%) and vas deferens (68.3 ± 4.2%). Moreover, no difference was observed between the results of viability and membrane integrity assessed by fluorescent probes techniques (*P* > 0.05).

## Discussion

In addition to the lack of information about the reproductive aspects of rheas, there is also a lack of validated methods for analyzing their sperm parameters. Therefore, this present study innovates by establishing efficient methodologies for the functional analysis of rhea spermatozoa along the spermatic pathway. We also highlight that the establishing of methods for obtaining, analyzing and processing spermatozoa directly from the spermatic pathways of valuable wild birds, in particular those accidentally killed, seems to be an additional option for the preservation of this valuable genetic material.

It is well known that different factors, such as the size of the epididymis and the diameter of the vas deferens, influence the success of the sperm retrieval technique (Chatdarong et al., 2010). Although the vas deferens is known as the storage site for sperm in birds (Tingari, 1971), the number of sperm retrieved by floating at the present study was lower than those previously obtained through cloacal massage in the same species, 2.24 x10^9^ spermatozoa (Góes et al., 2010). Anyway, since there were no differences in the quantity or quality of spermatozoa recovered from the epididymis to the vas deferens of the rhea, both sites could serve as a source of spermatozoa to be recovered and used for the formation of biobanks. Special care in processing only needs to be conducted with samples recovered from the epididymis, as these may be contaminated with blood cells. In fact, this finding is also described for some mammalian species (Bezerra et al., 2014), and sample centrifugation is recommended, since the presence of red blood cells could impair sperm quality and freezeability (Rijsselaere et al., 2004). To reduce the risk of contamination of blood cells, epididymal sperm collection by retrograde washing could be an alternative (Bezerra et al., 2014); however, when we tried to perform it previously, we were unable to cannulate the vas deferens for the addition of the solution that should drag the spermatozoa along the spermatic pathways. This was mainly due to the small caliber and tortuous course of these spermatic pathways (Santos et al., 2011). Thus, future investigation of cell separation techniques such as centrifugation or density gradient is recommended for future manipulations of spermatozoa from the epididymis of rheas.

The evaluations carried out by CASA are more reliable, objective and sensitive in detecting subtle motility characteristics, since the equipment evaluates cells individually (Verstegen et al., 2002). For birds, the use of this tool is important because it reduces the variations observed in the subjective evaluations carried out by direct microscopic observation as observed for *Amazona ventralis* (33%–82%) (Brock, 1991) and *Aratinga auricapilla* (39%–54%) (Stelzer et al., 2005). In our study, we initially tried to use the emu’s set-up settings for CASA previously stablished by Sood et al. (2012); however, we realized that many cells were not counted by the system. We attribute this mainly to the fact the head dimensions of rhea sperm (acrosome length = 0.97 ± 0.01 μm and head length = 7.54 ± 0.02 μm - Bezerra et al., 2023a) are lower than those verified for the emu (acrosome length = 1.84 ± 0.31 μm and head length = 11.77 ± 0.93 μm – du Plessis and Soley, 2014). Thus, considering that CASA assessments are based on head movement (Verstegen et al., 2002), modifications to the baseline configuration of emus were necessary to establish the best settings for the analysis of sperm from rhea cells. Thus, the kinematic data reported in the present study will serve as a basis for future research in rheas.

Our findings regarding sperm kinetic parameters differed from those reported for other ratites such as ostrich (Muvhali et al., 2022). However, it is worth mentioning that the evaluation may vary from one laboratory to another, factors such as sample dilution and preparation, the type of slide used, different versions of the computerized analysis system, can contribute to different results. For example, in ostriches, Muvhali et al. (2022) used the Sperm Class Analyzer^®^ (SCA) version 5.3 (Microptic S.L., Barcelona, Spain) with a Basler A312fc digital camera (Basler AG, Ahrensburg, Germany), mounted on an Olympus BX41microscope (Olympus Optical Co., Tokyo, Japan), different from the system used in our work. In addition, we also attribute the differences in results to the different origin of the samples, since our samples came from the ducts and epididymis and the ostrich samples were ejaculated.

Regarding sperm plasma membrane functionality, even if no difference was observed among the results found at the use of 0, 50 and 100 mOsm/l solutions, we believe that distilled water at 0 mOsm/l is the best option for this test. It is worth mentioning that distilled water has the advantages of being simpler, relatively cheaper and more accessible to handle than sugar-based solutions (Quintela et al., 2010). In fact, there is great variation regarding the most suitable solution for HOST among different species, since a 50 mOsm/L solution is indicated for emu (Matson et al., 2009), while a 100 mOsm/L solution has been suggested for the black castellana roosters (*Gallus gallus domesticus*) (Santiago-Moreno et al., 2009). Such different responses to HOST could be related to the fact that their gametes have different physical and biochemical properties, causing changes in the degree of penetration of electrolytes and non-electrolytes in their membranes (Vazquez et al., 1997).

The assessment of sperm viability using smears stained with vital dyes observed under light microscopy are still important and widely used for sperm analysis of various bird species (Sood et al., 2012; Fattah et al., 2017; Fischer et al., 2020), especially because they are cheap, fast and practically applied in the field. Although both vital dyes (bromophenol blue and eosin-nigrosine) were efficient for the evaluation of rhea sperm viability, visualization in eosin-nigrosine smears was better, easier and clearer than using bromophenol blue staining. We emphasize, however, that the use of fluorescent probes for membrane integrity analysis should be conducted always as possible, mainly because the sperm marking makes the evaluations more reliable than those conducted under the use of vital dyes (Arruda et al., 2011). Regarding the choice of fluorophores, it is worth mentioning that during the research an attempt was also made to evaluate the rhea sperm using propidium iodide combined with 6-carboxyfluorescein diacetate – CFDA (Peixoto, 2010), but this association was not efficient enough because cells marked with CFDA emitted fluorescence for only a short period of time, rapidly losing contrast with the bottom of the slide, and making visualization difficult. In contrast, Hoechst 33342 was able to emit fluorescence for a longer time and the contrast with the bottom of the slide was better. Hoechst 3342 is a fluorescent probe that penetrates the plasma membrane and binds preferentially to the adenine-thymine (AT) regions of DNA when excited by ultraviolet light it emits blue fluorescence (Sandhu et al. 1985). Hoechst 33342 and 33258 are the most commonly used bis-benzimides with similar excitation spectra; however, the former is significantly more permeable than the latter and therefore generally used to label cells (Bucevičius et al. 2018). PI has DNA affinity and marks red in the nuclei of cells with damaged plasma membranes and binds DNA by intercalation between bases with little or no sequence preference (Chalah and Brillard 1998). In this way, Hoechst has the ability to pass through cell membranes to mark the DNA of both living and dead cells, acting as a countermarker. On the other hand, PI can only penetrate through injured plasma membranes, promoting a selective labeling of damaged or dead cells.

Following a flash-frozen assay, the number of rhea cells with damaged membranes significantly increased from T100 to T50 and T0. This comparison was important to determine the efficiency of treatments prepared with deep-frozen samples. In fact, the marking patterns found for rhea sperm were similar to those previously described for quails (T100 = 62.2 ± 5.2%, T50 = 29.0 ± 4.1% and T0 = 0.1% ± 0.1%) (Bezerra et al., 2023b) and roosters (T100 = 86.29 ± 8.4% T50 = 46.40 ± 8.6% and T0 = 3.71 ± 2.8%) (Celeghini et al., 2007).

Although Bezerra et al. (2023a) had recently reported an increase in rheas’ sperm size along the spermatic pathways, our results showed that there appear to be no significant modifications in rheas’ sperm kinematic patterns and plasma membrane functionality and integrity, as detected by the methods used. In mammals, spermatozoa are known to undergo functional maturation during epididymal transit (Cornwall and Hann, 1995). This process involves various structural modifications, alterations in the lipid and protein compositions of plasma membranes (Jones, 1998) and development of motility (Morton et al., 1978). On the other hand, in birds, it was a long-standing dogma that there is no maturation process during epididymal transit in avian sperm due to epididymal regression. However, recent studies suggest that avian sperm gradually acquire the skills necessary for fertilization during their passage through the male genital tract prior to ejaculation (Ahammad et al., 2011).

In fact, the process of sperm maturation during its passage from the epididymis to the vas deferens in birds remains to be better investigated. Studies related to transcriptomic analysis of the reproductive tract in turkeys have identified some candidate genes that may be potentially involved in post-testicular sperm maturation, being differentially expressed in the epididymis and vas deferens (Słowińska et al., 2020). In this species, the achievement of sperm motility during post-testicular maturation appears to be associated with the development of flagellar actin filaments, with Ca 2+ influx and protein phosphorylation/dephosphorylation processes being the main regulatory mechanisms. Furthermore, sperm quality appears to be controlled by apoptosis and antioxidant systems of the epididymis and vas deferens (Słowińska et al., 2020). Another sign of sperm maturation in birds was demonstrated by Bernal et al. (2022) by demonstrating that during transit through the sperm canals, sperm chromatin increases the degree of compaction. In vitro experimental findings infer that avian spermatozoa undergo a gradual process of maturational changes in motility, acrosomal proteolytic activity and penetrability as a means of acquiring potential fertility during their passage through the male genital tract (Ahammad et al., 2011). None of this information, however, has been investigated in rheas to date, making clear the need for continued studies in an attempt to elucidate their reproductive phenomena, especially those related to sperm maturation.

Due to the low availability of animals for ethical reasons, our study was carried out with a limited number of animals. However, we emphasize that due to the lack of studies on this important species, our data are relevant. Reduced numbers of animals have also been reported in studies on other wild birds, even for least concerned species as the Eurasian hobby (*Falco subbuteo)* and the common krestel (*Falco tinnunculus* – Dogliero et al., 2016).

## Conclusions

In summary, we presented innovative information related to the motion kinematic patterns of rhea sperm along the spermatic pathways, providing a set-up adjusted for the species. Moreover, we suggest the use of distilled water as the hypoosmotic solution for the evaluation of membrane integrity of rhea sperm. Moreover, the use of bromophenol blue and eosin-blue dyes for assessing sperm viability was demonstrated as a practical and effective assay. Besides it, we indicate the use of the combination of fluorophores propidium iodide and Hoechst 33342 for the evaluation of rhea plasma membrane integrity. Finally, rhea sperm present similar motion kinematic patterns and plasma membrane functionality and integrity from the epididymis to the vas deferens. This set of information is valuable for expanding knowledge about the reproductive physiology of this near threatened species and is even applicable for the development of technologies for the conservation of its male gametes.
